# Mercury immune toxicity in harbour seals: links to *in vitro *toxicity

**DOI:** 10.1186/1476-069X-7-52

**Published:** 2008-10-29

**Authors:** Krishna Das, Ursula Siebert, Audrey Gillet, Aurélie Dupont, Carole Di-Poï, Sonja Fonfara, Gabriel Mazzucchelli, Edwin De Pauw, Marie-Claire De Pauw-Gillet

**Affiliations:** 1 Laboratoire d'Océanologie, Centre de Recherche MARE, B6C, Université de Liège, 4000, Liège, Belgium; 2 Research and Technology Center Westcoast, University of Kiel, 25761 Buesum, Germany; 3 GKSS Research Centre, Institute for Coastal Research, 21502, Geesthacht, Germany; 4 Laboratoire de Spectrométrie de Masse, B6C Liège, Université de Liège, 4000, Liège, Belgium; 5 Laboratoire d'Histologie et de Cytologie, B6C, Université de Liège, 4000, Liège, Belgium

## Abstract

**Background:**

Mercury is known to bioaccumulate and to magnify in marine mammals, which is a cause of great concern in terms of their general health. In particular, the immune system is known to be susceptible to long-term mercury exposure. The aims of the present study were (1) to determine the mercury level in the blood of free-ranging harbour seals from the North Sea and (2) to examine the link between methylmercury *in vitro *exposure and immune functions using seal and human mitogen-stimulated peripheral blood mononuclear cells (T-lymphocytes).

**Methods:**

Total mercury was analysed in the blood of 22 harbour seals. Peripheral blood mononuclear cells were isolated from seals (n = 11) and from humans (n = 9). Stimulated lymphocytes of both species were exposed to functional tests (proliferation, metabolic activity, radioactive precursor incorporation) under increasing doses of methylmercury (0.1 to 10 μM). The expression of cytokines (IL-2, IL-4 and TGF-β) was investigated in seal lymphocytes by RT-PCR and by real time quantitative PCR (n = 5) at methylmercury concentrations of 0.2 and 1 μM. Finally, proteomics analysis was attempted on human lymphocytes (cytoplasmic fraction) in order to identify biochemical pathways of toxicity at concentration of 1 μM (n = 3).

**Results:**

The results showed that the number of seal lymphocytes, viability, metabolic activity, DNA and RNA synthesis were reduced *in vitro*, suggesting deleterious effects of methylmercury concentrations naturally encountered in free-ranging seals. Similar results were found for human lymphocytes. Functional tests showed that a 1 μM concentration was the critical concentration above which lymphocyte activity, proliferation and survival were compromised. The expression of IL-2 and TGF-β mRNA was weaker in exposed seal lymphocytes compared to control cells (0.2 and 1 μM). Proteomics showed some variation in the protein expression profile (*e.g*. vimentin).

**Conclusion:**

Our results suggest that seal and human PBMCs react in a comparable way to MeHg *in vitro *exposure with, however, larger inter-individual variations. MeHg could be an additional cofactor in the immunosuppressive pollutant cocktail generally described in the blood of seals and this therefore raises the possibility of additional additive effects in the marine mammal immune system.

## Background

Mercury (Hg) is a widely present metal in the environment, with a major natural source being provided by degassing from the Earth's crust [1,2]. Its environmental level has also increased as a consequence of discharge from various industries, from medical and scientific waste, and from the processing of raw ores [1,2]. Following the discovery in the early 1960s of the dangers to human health of Hg in the marine environment, there has been a steady reduction in the man-made discharge of Hg [3]. However, despite these regulations, a decrease in levels of mercury in the biota, including marine mammals, is not obvious [3-6].

Methylation of inorganic Hg is a key processes in marine food webs affected by several variables including temperature [7-10]; It raises serious concerns in the light of global change and increasing seawater temperature [9,10]. MeHg has a high bioavailability; it bioaccumulates and biomagnifies at all trophic levels in the food web and has severe toxicological effects [11,12]. Fish represent the major MeHg source for human and marine mammal populations [13-17].

High Hg concentration have been documented in the liver and kidney of marine mammals (from both pristine and contaminated areas), generally associated to Se in a non organic form (tiemannite or HgSe) [18-21]. The high relevance of tiemannite precipitation in marine mammals (compared to other mammals and birds) is likely to be related to a combination of factors, namely, elevated MeHg exposure, due to fish eating habits, and the inability to excrete MeHg through gills, feathers or fur [21-24].

However, the demethylation process is not "instantaneous" and before reaching these long-term accumulation organs, Hg is assimilated from fish, and is transferred and transported via the blood stream, mainly in its methylated form [11,12]. A high percentage of blood Hg concentration is in a methylated form (up to 90%), especially in human and marine mammal populations relying on fish [11,12,25] and so may represent a threat towards blood cells, including immune cells.

It has been suggested that impairment of immune function plays a contributing role in the increasing incidence of infectious diseases in marine mammals. Moreover, adverse effects of environmental contaminants on the immune system have often been suggested [26-29]. Information on the immune system of harbour seals is quite well documented. This species has conveniently become the marine mammal of choice for immunological studies [27,28]. Interest in the harbour seal has stemmed partly from earlier captive studies on the reproductive toxicity of environmental contaminants using this species [30], but more importantly this interest has occurred as a consequence of recurrent phocine distemper virus (PDV) epizootics [31-33]. However, despite numerous studies involving the *in vivo *and *in vitro *effects of persistent organic pollutants [29,34-44], information on the effects of Hg on the marine mammal immune system and underlying mechanisms remains scarce [45-48].

The aims of the present study were (1) to determine T-Hg levels in the blood of free-ranging harbour seals from the North Sea and (2) to examine the link between *in vitro *Hg exposure at low doses and immune functions using seal and human mitogen-stimulated peripheral blood mononuclear cells (PBMCs). Seals and humans were exposed *in vitro*, using various MeHg concentrations (0.1 to 10 μM), reflecting the levels encountered in wildlife. MeHg was chosen as it is the predominant form in the blood of marine mammals and fish-eating communities [12,17,49]. We assessed the effects of MeHg on T-cell viability and the proliferative response to mitogen of peripheral lymphocytes in both harbour seals and humans. We also examined the effect of MeHg on the *in vitro *production by seal PBMCs of cytokines IL-2, IL-4 and TGF-β(0.2 and 1 μM). Finally, we attempted through the use of proteomics analysis, to delineate the biochemical pathways of MeHg in *in vitro *exposure (1 μM) using a first human PBMC model.

## Methods

### Blood sampling

Blood was sampled from 33 harbour seals caught along the German coast or kept in captivity (Seal Station, Friedrichskoog, Germany) between 1997 and 2006 (Table 1). The seals were physically restrained. Health status was determined by physical examination, using routine haematological analysis (blood counts) and serum chemistry (detailed protocol described previously [50]).

**Table 1:** Sampling description of harbour seal *Phoca vitulina*

**ID**	**Date**	**Sampling site**	**Sex**	**Length (cm)**	**Body mass (kg)**	**Assay**
**Pv 455**	20/10/97	Lorenzensplate	M	117	32	T-Hg
**Pv 456**	20/10/97	Lorenzensplate	F	104	30	T-Hg
**Pv 460**	20/10/97	Lorenzensplate	M	133	49	T-Hg
**Pv 2257**	09/04/03	Lorenzensplate	M	144	68	T-Hg
**Pv 2258**	09/04/03	Lorenzensplate	M	148	63	T-Hg
**Pv 2259**	09/04/03	Lorenzensplate	F	129	38	T-Hg
**Pv 2260**	09/04/03	Lorenzensplate	F	143	48	T-Hg
**Pv 2261**	09/04/03	Lorenzensplate	F	158	56	T-Hg
**Pv 2262**	09/04/03	Lorenzensplate	F	132	46	T-Hg
**Pv 2263**	09/04/03	Lorenzensplate	F	148	55	T-Hg
**Pv 2265**	09/04/03	Lorenzensplate	F	148	55	T-Hg
**Pv 2687**	25/08/04	Lorenzensplate	M	170	84	T-Hg
**Pv 2688**	25/08/04	Lorenzensplate	M	170	82	T-Hg
**Pv 2689**	25/08/04	Lorenzensplate	M	175	80	T-Hg
**Pv 2690**	25/08/04	Lorenzensplate	M	175	90	T-Hg
**Pv 2691**	25/08/04	Lorenzensplate	F	150	58	T-Hg
**Pv 2692**	25/08/04	Lorenzensplate	F	165	70	T-Hg
**Pv 2694**	25/08/04	Lorenzensplate	M	160	75	T-Hg
**Pv 2695**	25/08/04	Lorenzensplate	M	175	87	T-Hg
**Pv 2697**	25/08/04	Lorenzensplate	F	135	42	T-Hg
**Pv 2699**	25/08/04	Lorenzensplate	M	140	40	T-Hg
**Pv 2700**	25/08/04	Lorenzensplate	M	125	39	T-Hg
**Kirsa**	28/02/05	*Seal station*	F	nd	28	Cytokine
**Pv 2883**	12/04/05	Lorenzensplate	M	174	93	Cytokine
**Pv 2885**	12/04/05	Lorenzensplate	M	168	81	Cytokine
**Pv 2887**	12/04/05	Lorenzensplate	M	180	96	Cytokine
**Pv 2893**	12/04/05	Lorenzensplate	M	164	71	Cytokine
**Lümmen**	31/03/2006 30/07/2006	*Seal station*	M	nd	105	Functional tests
**Hein**	31/03/2006 30/07/2006	*Seal station*	M	nd	86	Functional tests
**Deern**	31/03/2006	*Seal station*	F	nd	70	Functional tests
**Lilli**	28/04/2006	*Seal station*	F	nd	54	Functional tests
**Mareike**	28/04/2006	*Seal station*	F	nd	66	Functional tests
**Kirsa**	28/04/2006	*Seal station*	F	nd	35	Functional tests

Blood was drawn from the extradural venous sinus into sterile evacuated blood collection Monovette^® ^tubes (serum tubes for Hg analysis, EDTA tubes for RT-PCR and heparinised tubes for functional tests and proteomics analysis). Blood tubes were kept at -20°C until Hg analysis or were processed within 18 hours for cell cultures.

Buffy coats from healthy 30–60 year old male humans (n = 9) were integrated into this study for the PBMC culture (Croix Rouge de Belgique).

### Mercury analysis

The measurement of the concentration of T-Hg in blood is generally a good surrogate for the concentration of MeHg in blood in human populations with a high fish consumption [12,16]. T-Hg was analysed in full blood from 22 harbour seals by cold vapour atomic absorption spectrometry on a Perkin-Elmer Coleman Mas-50 Mercury Analyser (wavelength 253.7 nm). The freeze-dried samples were subjected to microwave-assisted digestion with nitric acid and H_2_O_2_, as described previously for blood and other tissues [51,52]. Quality control measurements for T-Hg included replicate analysis resulting in coefficients of variation < 10% and analysis of certified material (DORM-1, NRC, Canada). The Hg absolute detection limit was 10 ng, corresponding to 0.13 μg.g^-1 ^fresh weight (fw) for an average of 1.5 g of sample analysed. All samples were above the detection limit.

### Cell cultures

#### Harbour seals

The blood was diluted 1:2 with phosphate-buffered saline (PBS). Lymphocytes were separated on a Ficoll gradient (Pashing and Amersham), washed twice in PBS and suspended in culture medium containing 10% foetal calf serum (Minimum essential Medium, Eagle for cytokine expression and RPMI 1640 Cambrex added with 1% L-glutamine and 10% penicillin-streptomycin for functional tests). PBMCs were incubated without mitogen or in the presence of phytohemaglutinin (PHA, 5 μg.ml^-1^), a T-cell-specific mitogen [42,53]. 2 × 10^5 ^cells were seeded in a final volume of 300 μl per well (for cytokine expression) or 100 μl per well (functional tests). To some of these wells, CH_3_HgCl (Sigma-Aldrich) was added in different concentrations (0.1 to 10 μM depending on the assay). Cells were then incubated for 72 h at 37°C with 5% CO_2_.

#### Humans

PBMCs were isolated from buffy coats by using standard Ficoll/Hypaque (Amersham) gradients and were washed twice in PBS. The pellet was suspended in the culture medium (RPMI 1640 supplemented with 10% heat inactivated foetal bovine serum (in vitrogen), 1% L-glutamine, 10% penicillin-streptomycin; Cambrex). 2 × 10^5 ^cells were seeded in 100 μl and were incubated in a 96-well microculture plate (Falco, New Jersey, USA). For proteomics analysis, 20 ml were deposited in T75 flasks (Susp Cell Ven green, Sarsted). PHA was diluted in culture medium at an optimal concentration of 1 μg.ml^-1 ^[54].

### Cytokine expression

The expression of the housekeeping gene glyceraldehyde-3-phosphate (GAPDH), of interleukins-IL-2 and IL-4 and of the transforming growth factor (TGF)-β was investigated *in vitro *in control and contaminated seal PBMCs (n = 6, Table 1) using RT-PCR and real time PCR, as described elsewhere [53,55,56]. For a quantitative analysis of mRNA expression, a comparison between samples can be made by relating gene expression levels to housekeeping gene expression, the latter regarded as being unaffected by exposure conditions. After the incubation period (72 h), total RNA was isolated from exposed and control PBMCs (RNeasy^® ^Mini Kit, Qiagen Sciences, Maryland, USA) following the manufacturer's recommendations. After DNase-treatment (DNA-*free*™, Ambion kit), RNA was reverse transcribed with murine reverse transcriptase (RNA PCR Core Kit™, Applied Biosystems, Weiterstadt, Germany) and the resulting cDNA served as a template for PCR following the manufacturer's protocol and using Thermocycler MX4000™ (Stratagene Europe). For real-time quantification, the Brilliant SYBRGreen QPCR Master mix (Stratagen Europe) was used [56]. This contained SYBRGreen I as a fluorescence dye, dNTPs, MgCl_2 _and a hot start Taq DNA polymerase. Primers for the PCR were designed using DNAstarTM software (GATC Biotech, Konstanz, Germany). Primers for the detection of IL-2 and IL-4 were selected from conserved nucleotide sequences of grey seals (*Halichoerus grypus*) and dogs (*Canis familiaris*) respectively (GenBank accession numbers AF072871, AF187322, AF104245). Primer sequences for the amplification of GAPDH and TGF-β were selected from previously published sequences [53] (Table 2).

**Table 2:** Primer sequences used for the amplification of cytokine and housekeeping gene transcripts in lymphocytes

Gene	**Primer sequence (5'-3')**	**Direction**	**Annealing temperature**	**Nucleotide position**	**Calculated length of amplicon**	Sequences used for primer pair selection (accession number, NCBI)
IL-2	TTT AAG TTT TAC ACG CCC AAG TGT TTC AGA TCC CTT TAG TTTC	S AS	55°C	218–400	183 pb	AF072871
IL-4	ACT CAC CAG CAC CTT TGT CCA TCC TTA TCG CTT GTT CTT TG	S AS	49°C	48–200	153 pb	AF187322, AF104245
TGF-β	TTC CTG CTC CTC ATG GCC AC GCA GGA GCG CAC GAT CAT GT	S AS	57°C	826–845 1218–1199	393 pb	[51]
GAPDH	GCC AAA AGG GTC ATC ATC TC GGG GCC ATC CAC AGT CTT CT	S AS	57°C	1225–1244 1452–1433	228 pb	[51]

bp = base pair; S = sense; AS = antisense; IL = interleukin; TGF = transforming growth factor

The fluorescence response was monitored in a linear fashion, as the PCR product was generated over a range of PCR cycles. For each cytokine, a standard curve was prepared using a dilution series from 10^9 ^to 10^2 ^copies. The PCR started with an initial step at 95°C for 10 min, followed by 40 cycles with denaturation at 95°C for 1 min, annealing temperature for 30 sec and elongation at 72°C for 1 min. The fluorescence was measured at the end of the annealing and at the end of the dissociation program at a wavelength of 530 nm. In order to exclude measurement of non specific PCR products and primer dimers, and to determine true amplification, each PCR was followed by a dissociation program for 1 min at 95°C, followed by 41 cycles during which the temperature was increased in each cycle, starting at 55°C and ending at 95°C. Only PCR reactions with one well-defined peak were used for analysis. All reactions were performed in duplicate and two separate PCR reactions were performed. GAPDH was used as the control gene. In order to calculate cytokine expression, GAPDH was used as the calibration compound. The cytokine expression index (CI) was calculated as follows:

*CI = Number of cytokine copies/Number of GAPDH copies*

### Viability of PBMCs and functional tests

The viability and number of PHA-stimulated PBMCs were determined microscopically by trypan blue exclusion assay and a haemocytometer before and after MeHg exposure.

### MTS assay

After 72 hours of MeHg exposure (0.1, 0.2, 0.5, 1, 1.5, 5, 10 μM), the metabolic activity of the cells was quantified by a colorimetric microtitre plate MTS assay 3-(4,5-dimethylthiazol-2-yl)-5-(3-carboxymethoxyphenyl)-2-(4-sulphophenyl)-2H-tetrazolium, inner salt (MTS, Promega), according to the manufacturer's instructions. 10 μl of prepared MTS solutions were added to the 100 μl culture medium containing the cells. MTS is chemically reduced by cells into formazan, which is soluble in culture medium. After 2 h in the dark and in the incubator, the measurement of the absorbance of the formazan was carried out on a spectrophotometer (Powerwave X, Bio-Tek) at 492 nm. Each condition was produced in 5 wells.

### Incorporation of radioactive precursors

DNA, RNA and protein synthesis were determined by measuring the amount of incorporated radioactive precursors using a scintillation counter. 2 × 10^5 ^cells were seeded in 200 μl culture medium. During the last 24 h of culture, 1 μCi.ml^-1 ^of methyl-[^3^H] thymidine (specific activity 20–40 Ci.mmole^-1^), [^3^H] uridine (specific activity 20–40 Ci.mmole^-1^) or [^3^H] L-leucine (specific activity 40–60 Ci.mmole^-1^, MP Biomedicals) was added to each well and cellular incorporation was determined. Cells were harvested on a filter (Multiscreen HTS, Millipore) with a millipore aspiration system, as described by the manufacturer's instructions. The following steps were carried out with an automatic dispenser (Precision Power 2000): 100 μl PBS, 120 μl trypsin 10 min, 100 μl PBS, 100 μl ethanol 20 min, 3× 100 μl ethanol. The plates were dried overnight, and the filters were collected in vials (Puncher Millipore). 400 μl sodium hypochlorite (1:10 v:v) were then added. After 30 min agitation, 4 ml scintillating liquid (Ready Safe, Beckman) were added and quantification of the retained radioactivity by the cells was made by liquid scintillation in a counter (LS 6500 Scintillation system, Beckman). Results from triplicates (means of counts per minute ± standard deviations) are expressed in percentages (control taken to be 100%).

### Proteomics analysis

Preliminary proteomics analysis (the study of the proteome) was carried out on human PBMCs. We focus this part of the study on cytoplasmic fraction (soluble proteins). Cells were treated with 1 μM MeHgCl in the presence of PHA for 72 h. Quantitative analysis was carried out on 2D DIGE gels in two steps. First, individuals were treated separately (n = 3) in order to discover interindividual variability and secondly, individuals were pooled in order to obtain sufficient amount of proteins to run the gel.

#### Protein preparation

After 72 h of MeHg exposure (1 μM), cells were harvested with PBS. Subcellular perfractionation was performed to isolate cytosolic proteins, as described previously [57]. Cytosolic proteins were extracted in a hypotonic buffer (10 mM HEPES, pH 7.4; 10 mM NaCl; KH_2_PO_4 _1 mM, NAHCO_3 _5 mM, EDTA 5 mM, CACl_2 _(H_2_O)_2 _1 mM, MgCl_2 _(H_2_O)_6 _0.5 mM and EDTA-free antiproteases (Roche Molecular Biochemicals) followed by centrifugation. Pellets were suspended in isotonic buffer (sucrose, 2.5 M) before two centrifugation steps at 6300 g (elimination of nucleus and large size cellular fragments) and 107,000 g (elimination of organites and membranes). The different extracts were purified using the 2D Clean-Up Kit (according to the manufacturer's instructions). After this purification step, each sample was redissolved in the DIGE buffer (urea 7 M, thiourea 2 M, Tris-HCl pH 8.8 30 mM, Chaps 1.5%, ABS-14 1.5%). After a second assay, allowing us to discover the exact concentration of our extracts, the samples were diluted to a protein concentration of 5 μg.μl and to pH 8, using DIGE buffer. A Mowse score (MOlecularWeightSEarch) was calculated, based on peptide frequency distribution [58].

#### Protein separation

After extraction, the variations in protein abundance between the treated (with 1 μM of MeHg) and non-treated samples were measured using the 2D DIGE technique (Two Dimension Difference Gel Electrophoresis).

The labelling of both the control fraction (12.5 μg), and the exposed fractions (12.5 μg) was carried out with 100 pmol of Cye 3 and of Cye 5 respectively. The internal standard consisted of a mixture of each sample (12.5 μg per sample) labelled with 1200 pmol of Cye 2. This step was then followed by incubation on ice for 45 minutes in the dark. The eventual excess of Cye was eliminated by the addition of 10^-4 ^mol of lysine on ice for 15 minutes in the dark. The labelled samples were pooled. In addition 150 μg of unlabelled protein were added to achieve a sufficient quantity of unlabelled protein required for analysis by mass spectrometry. A volume of buffer 2× (urea 7 M, thiourea 2 M, chaps 1.5%, ASB-14 1.5%, destreak reagent 12.5 μl.ml^-1^, DTT 10 mM, ampholyte 1%) was added in equivalent quantities to the previously pooled samples. The final volume of the samples was brought up to 450 μl with IEF buffer (urea 7 M, thiourea 2 M, chaps 1.5%, ASB-14 1.5%, destreak reagent 12.5 μl.ml^-1^, ampholyte 0.5%, bromophenol blue 0.5%).

The measurement of the first dimension of the gel was carried out on gel strips with a fixed pH gradient (Immobiline DryStrip) in an electrical focalisation cell (Protean IEF cell, Bio-Rad). After active rehydration for 9 h with the sample, isoelectric focalisation was carried out up to 50,000 Vh overnight. Before the second dimension was measured, reduction buffer was added for 15 minutes (DTT 130 mM, urea 6 M, Tris-HCl pH 8.8 0.373 M, glycerol 20%v/v, SDS 2%w/v) followed by the addition of an alkylation buffer for 15 minutes (iodoacetamide 135 mM, urea 6 M, Tris-HCl pH 8.8 0.373 M, glycerol 20%v/v, SDS 2%w/v).

The measurement of the second dimension was carried out overnight after depositing the strips on a 12.5% polyacrylamide gel (Tris-HCl pH 8.8 1.5 M, SDS 0.4%w/v, acrylamide/bisacrylamide 40%, ammonium persulphate 10%w/v, TEMED 0.03%v/v, MilliQ water). The gels were then placed on non-fluorescing (Bind-Silane) plates (Ettan-DALT "Low Fluorescence" Casting Cassette, Amersham Biosciences). After migration, the gels were scanned at the three different wave lengths of the cyanines (Cye2 520 nm bandwidth 40, Cye3 580 nm Bp 30, Cye5 670 nm Bp 30) using a Typhoon 9400 (Amersham Biosciences), with a resolution of 100 μm. The images obtained were analysed using a software program (Decyder 6.0, Amersham Biosciences). Only the regulations ± 1.5 above the standard deviation were taken into account and compared with a T Student value at a 95% confidence level.

#### Mass spectrometry protein identification

The spots of interest, selected using the software program were excised from the gel, by a spot picker (Ettan Spot Picker, Amersham Biosciences), and were then placed in 96 well plates for enzymatic digestion (Proteineer dp automated digester, Bruker). The peptides resulting from the digestion were spotted onto a prespotted MALDI plate (AnchorChips, Bruker). The proteins were identified using an Ultraflex MALDI TOF/TOF (Bruker). The mass values from the mass fingerprints, and from the MS/MS spectra were processed within different data bases (Sprot, NCBI, MSDB). The search engine used was MASCOT, using the following parameters: the mass tolerance for peptides was set at ± 60 ppm, the charge state at 1+ and the maximum number of missed cleavages at 1.

### Data analysis

Student's t-test was used for comparing control and MeHg-treated cells.

## Results

### Hg levels in the blood of free-ranging seals

**Figure 1:**
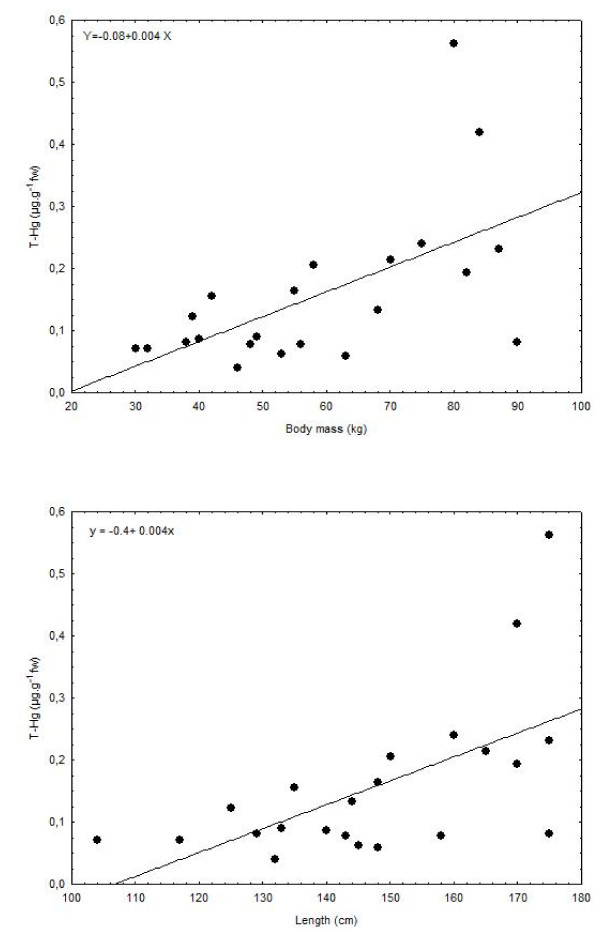
Relationship between body mass (graph on top) and length (graph on the bottom) and T-Hg concentrations in blood of harbour seals.

T-Hg levels in full blood varied from 0.04 to 0.56 μg.g^-1 ^fw (43 to 611 μg Hg.L^-1^) with a mean concentration of 0.16 μg.g^-1 ^fw (Table 3). T-Hg concentrations were found to be similar between males and females (Student's t-test, p > 0.5) and individuals were therefore regrouped. T-Hg concentration in blood was found to be significantly correlated to the body mass (r_p _= 0.59 and p < 0.001) and length of the seals (r_p _= 0.60 and p < 0.001) (Figure 1).

**Table 3:** Total Hg concentrations in blood of pinniped species

**Species**	**Location, year**	**Condition**	**Reference**	**Concentration**	
				**μg.L^-1^**	**μg.g^-1 ^fw**
				
***Phoca vitulina***	North Sea. (1997–2004)	Free-ranging seals	this work	172 ± 143 (43–611) n = 22	0.16 ± 0.13 (0.04 – 0.56) n = 22
***Phoca groenlandica***	NW Atlantic	Captive seal (before MeHg exposure)	[61]	80 ± 40 (n = 6)	nd
***Phoca groenlandica***	Gulf of St Lawrence (1976 – 1978)	Free-ranging adult seals	[62]	nd	Males: 0.15 ± 0.02 n = 2 Females: 0.07 ± 0.04 n = 48
***Phoca groenlandica***	Front ice off Newfoundland – Labrador (1976–1978)	Free-ranging adult seals	[62]	nd	Males: 0.04 ± 0.02 n = 3 Females: 0.02 n = 1
***Leptonychotes weddellii***	Antarctic 1980	Free-ranging seals	[63]	nd	0.02 n = 2
***Callorhinus ursinus***	Alaska, 1972	Wild nursing cows	[85]	nd	0.099 n = 2

mean ± standard deviation, n = number of samples analysed, nd = not determined

### Proliferative response of controls and exposed human and seal PBMCs

**Figure 2:**
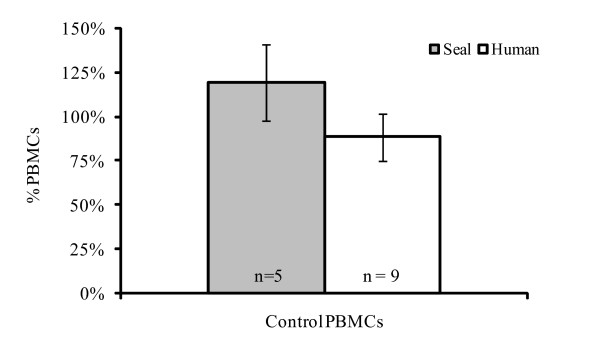
**Response of control PBMCs to PHA after 72 h of stimulation**. Results are expressed as a percentage expressed relative to the initial 2.10^6 ^cells per ml of culture medium. PHA concentration: 5 and 1 μg.ml^-1 ^for harbour seals and humans respectively.

**Figure 3:**
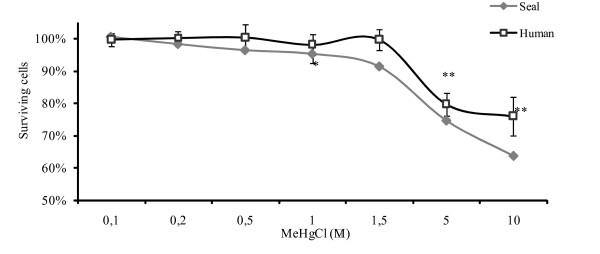
**Percentage of surviving cells estimated by Trypan blue coloration after 72 h of MeHg exposure**. * p < 0.05; **, p < 0.01 relative to control PBMCs 100%. 5 wells per condition for both species. Humans: n = 8 for 0.1 μM and 1 μM; n = 3 for other concentrations. Harbour seals: n = 5 for 1 μM and n = 2 for other concentration.

The PHA-induced proliferative responses of PBMCs collected from seals and humans are shown in Figure 2. A significant decrease in the percentage of lymphocyte proliferation was found at MeHg concentrations of 5 and 10 μM in both seals and humans (Figure 3). Cell mortality was found to be moderate at 1 and 1.5 μM of MeHg.

### Functional tests

**Figure 4:**
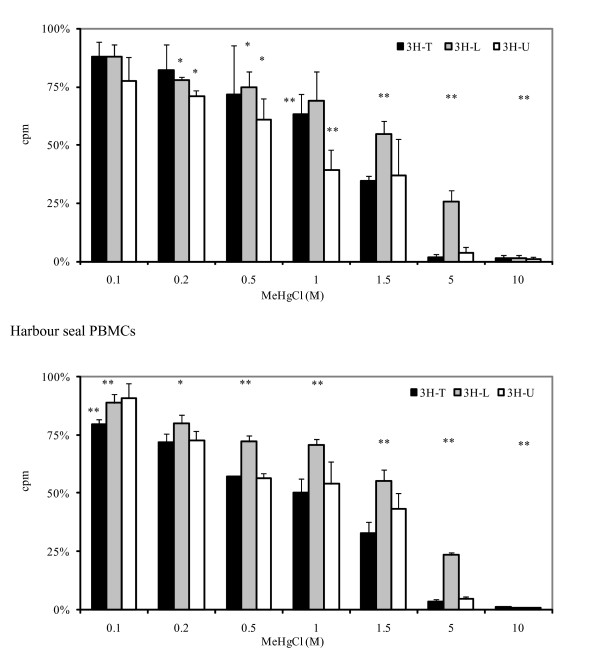
**Effects of MeHg exposure concentration on DNA (^3^H-thymidine), RNA (^3^H-uridine) and proteins (^3^H-leucine) in PBMCs**. Results from quadruplets (means of counts per minute ± standard deviation, n = 4) are expressed in percentages (control taken to be 100%).

A decrease in DNA, RNA and protein synthesis was observed even at low MeHg concentrations (0.2 and 1 μM), whereas inhibition of synthesis was clearly noticed at 10 μM (Figure 4). DNA, RNA and protein synthesis showed a similar profile but nucleic acid synthesis seemed more inhibited compared to proteins. After 48 h, the concentrations inhibiting 50% of DNA and RNA synthesis of the two species were found to be around 1 μM and 1.5 μM, while the 50% protein synthesis inhibiting concentration was found to be around 1.5 μM.

**Figure 5:**
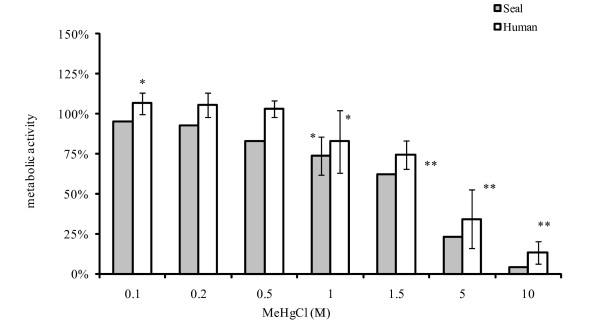
**MTS activity of human and seal PBMC relative to MeHg exposure**. * p < 0.05; **, p < 0.01 relative to control PBMCs 100%. For humans: n = 7 for 0.1 μM and 1 μM; n = 3 for other concentrations. For harbour seals: n = 5 for 1 μM and n = 2 for other concentrations. 5 wells per condition for both species.

The MTS assay (water soluble tetrazolium salts) reflects cell proliferation and the metabolic activity of the mitogen-stimulated lymphocytes. Metabolic activity was significantly reduced compared to the controls at around 1 μM both for human and seal cells (Figure 5).

### Detection of housekeeping gene and cytokines

**Figure 6:**
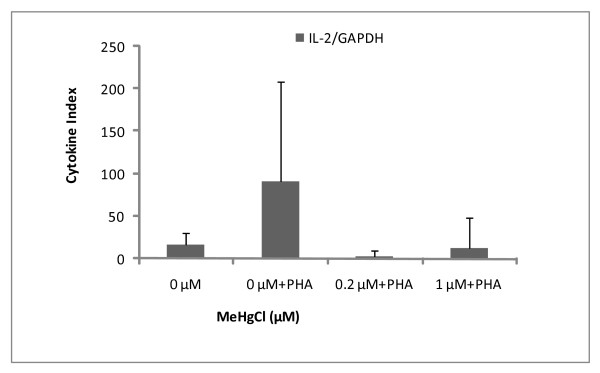
**Cytokine index of IL-2 mRNA in function of MeHg exposure**. Results are expressed in mRNA copy numbers per GAPDH mRNA copy. The mean and standard deviation on the mean of four individuals, carried out in duplicate, are shown.

**Figure 7:**
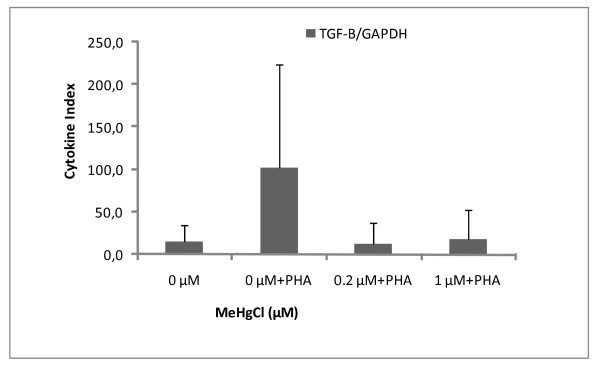
**Cytokine Index of TGF-β mRNA in function of MeHg exposure**. Results are expressed in mRNA copy numbers per GAPDH mRNA copy. The mean and standard deviation on the mean of four individuals, carried out in duplicate, are shown.

**Figure 8:**
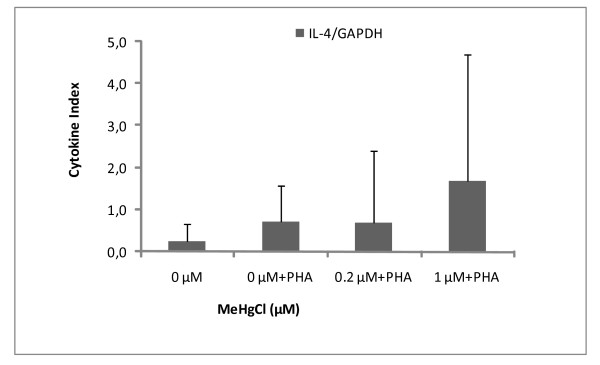
**Cytokine Index of IL-4 mRNA in function of MeHg exposure**. Results are expressed in mRNA copy numbers per GAPDH mRNA copy. The mean and standard deviation on the mean of four individuals, carried out in duplicate, are shown.

In this set of experiments, seal PBMCs were cultured in the presence of non-cytotoxic doses of MeHg (0.2 and 1 μM) in order to examine mRNA expression. GAPDH, IL-2, IL-4 and TGF-β mRNA were successfully detected in both controlled and exposed PBMCs (Figures 6, 7, 8). As a general feature, change in mRNA expression occurred after exposure: the number of copies decreased at 0.2 μM, except for IL-4. The large dispersion of data between seals (coefficient of variation ≤ 68%) indicated high grade inter-individual variability in cytokine mRNA responses. Cytokine indexes were strikingly low for IL-2 and TGF-β even at 0.2 μM (Figures 6 and 7), while an increasing trend was observed for IL-4 (Figure 8).

### Proteomics analysis

**Figure 9:**
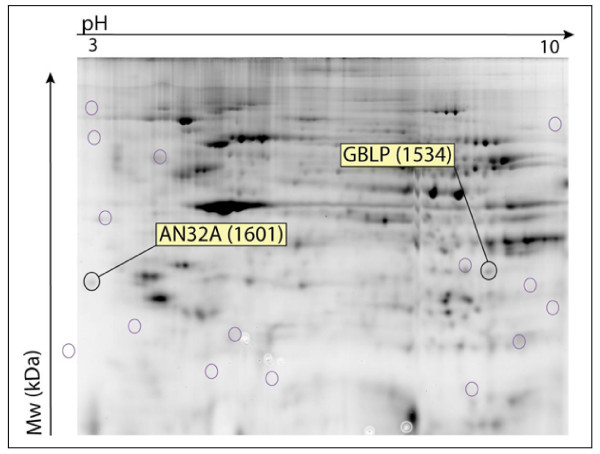
**2D-Dige Gel with cytoplasmic proteins extracted from one human control and treated PBMCs**. Each of the 3 individuals were analysed separately. O: excised spot for further protein identification. IPG strip pH 3 to 10.

**Figure 10:**
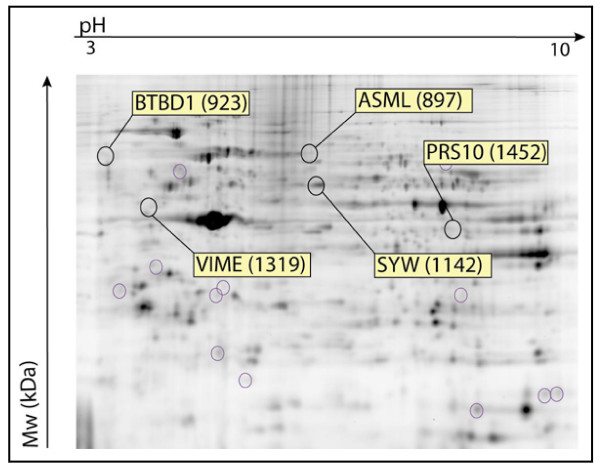
**2D-Dige Gel with cytoplasmic proteins extracted from human control and treated PBMCs**. Cytoplasmic proteins extracted from 3 individuals were pooled for this gel. O: excised spot for further protein identification. IPG strip pH 3 to 10.

Most of the proteins had an Ip of between 3 and 10. A Mowse score was calculated for each identification, which was considered as significant above 54 (Additional file 1). Low doses of MeHg (1 μM) were found to affect cytoplasmic protein expression. Proteins are generally less expressed in treated gels. Volume ratio (expression of protein in treated gel compared to control) was positive (over-expression) for spot 1142 and spot 1452 but negative (lower-expression) for spots 1319 (vimentin), 897 and 923 (Table 2, Figures 9 and 10). These results showed that the identified proteins are involved in many cellular functions such as cell proliferation (SYW), the building of the cytoskeleton (VIME), protein degradation (PRS10), melatonin biosynthesis and the creation of transduction pathways (GBLP, AN32A) (Additional file 1).

## Discussion

In the present study, T-Hg concentrations in the blood of harbour seals were found to vary widely, correlated to length and body mass of the seals (Figure 1). The observed correlation reflects daily Hg intake and thus, the amount of fish ingested, which differs according the body mass of animals. Adult harbour seals eat 5% to 6% of their body weight per day, up to 7 kg for big individuals [59,60]. T-Hg levels measured in the blood of harbour seals caught in the North Sea are higher than those previously described in North Atlantic or in Arctic regions (Table 3) [61-63]. Interestingly, the level of T-Hg in the blood of harbour seals from the North Sea is not lower than that encountered in other seal species 30 years ago. Similarly, Hg levels have not decreased in Arctic biota despite the recent reductions in emissions in North America and Western Europe [4-6].

A commonly used reference interval for human beings is 0.6 – 59 μg.L^-1^[64,65]. Clear signs of Hg toxicity develop in most individuals only at some point much higher than the upper reference limit. The Environmental Protection Agency (USA) has recommended that blood Hg levels should not be higher than 5.8 μg.L^-1^, at least for the more sensitive individuals such as pregnant women [17]. A study of a human cohort with high fish consumption in the Faroe Islands found a median cord blood concentration of 24 μg.L^-1 ^[66]. Obviously, the harbour seals we studied from the North Sea displayed higher concentrations (mean Hg concentration = 172 μg.L^-1^, around 1 μM). Knowing that Hg is mainly under a methylated form in the blood of marine mammals (up to 90%), questions arise regarding the potential biochemical effects of these Hg levels on harbour seals and human immune cells. To tentatively answer this question, a set of *in vitro *experiments were carried out on T-lymphocytes using low MeHg exposure (around 1 μM).

We exposed harbour seal and human lymphocytes *in vitro *to MeHg and we examined the effects on cell-mediated immunity: cell mortality, synthesis of DNA, RNA and protein and metabolic activity. Cell mortality was reduced in the interval 0.1–1 μM (Figure 3) both for human and seal PHA-stimulated PBMCs. Up to 90% of cells exposed to 1 – 1.5 μM of MeHg remained alive. However, a clear suppressive effect of MeHg on DNA, RNA and protein synthesis in seal and human PBMCs was found to be present, even at low concentrations. Protein synthesis seemed less affected, probably due to a longer response time (Figure 4). Proliferation and metabolic activity reflected by MTS assay confirmed that 1 μM was a critical concentration with significantly reduced *in vitro *activity of human and seal PBMCs relative to controls (Figure 5). No striking difference appeared between human and seal *in vitro *resistance to MeHg. At higher concentrations, 5–10 μM, 75% of cells remained alive, but metabolic activity dropped to very low levels.

Cytokine expression also decreased following MeHg exposure: IL-2 and TGF-β seemed highly sensitive to *in vitro *MeHg exposure in regard to their dramatic decrease in gene expression at 0.2 μM and 1 μM compared to controls (Figures 6, 7, 8). However, large inter-individual variability has previously been observed and cytokine expression in seal PBMCs has been found to vary widely, depending also on the duration of MeHg exposure [48]. A decrease in PBMC IL-2 expression has also been documented in harbour seals following *in vitro *exposure to PHA and PCB [37], raising the possibility of additive effects of MeHg and other organic pollutants.

IL-2 has a prime role in immune response, as it is responsible for T-cell clonal expansion after antigen recognition. IL-2 also increases synthesis of other T-cell cytokines, promotes the proliferation and differentiation of NK cells, and acts as a growth factor and stimulus for B-cell antibody synthesis. TGF-β is often considered as an anti-inflammatory cytokine [67,68]. A variety of murine models have provided evidence that eliminating TGF-β or disrupting its downstream signalling cascade leads to inflammatory disease [69].

In contrast, in the present study, the cytokine index of IL-4 showed an increasing trend from control to 1 μM (Figure 8). IL-4 is known to induce the differentiation of naive helper T cells (Th0 cells) into Th2 cells. Similarly, Devos et al. found that inorganic mercury (HgCl_2_) and MeHg was capable of increasing IL-4 production in Con A-stimulated human PBMC *in vitro *[70]. They also observed that IL-4 production occurred in a dose-dependent fashion, although MeHg was found to be much more potent than inorganic mercury in inducing IL-4 production [70]. Furthermore, they observed MeHg induced IL-4 production at a similar range of concentrations to those found in our own investigation (0.1–0.5 uM) [70].

These preliminary *in vitro *results suggest that MeHg could induce a differentiation of naïve cells (increase of IL-4), while T-lymphocyte clonal expansion is inhibited (decrease of IL-2); a decrease in TGF-β suggests an increase in inflammatory response and would require further investigation such as through a polynuclear cell model. Our findings are consistent with those of previous researchers working with human and rodent systems and support a hypothesis of contaminant-altered lymphocyte function mediated (at least in part) by the disruption of cytokine production (TH2 cells) [34,37,71,72]. Whether this phenomenon has clinical relevance in marine mammal populations remains to be determined.

### Study of the proteome

Proteomics facilitates the identification of new biomarkers of chemical exposure and studies of mechanisms by which protein modification contribute to the adverse effects of environmental exposure [73,74]. The proteome of T-lymphocytes is well known [75-77]. However, the expression of proteins in MeHg-exposed T-lymphocytes has not yet been described. Our results showed that identified proteins are involved in many cellular functions such as cell proliferation (SYW), the building of the cytoskeleton (VIME), protein degradation (PRS10), melatonin biosynthesis (ASML) and the creation of transduction pathways (GBLP, AN32A). Human lymphoid cells are an important physiological source of melatonin, which could be involved in the regulation of the human immune system [78]. As a general feature, many spots are underexpressed in exposed gels, reflecting the inhibition of protein synthesis linked to MeHg toxicity. As for cytokine mRNA expression, high variability between individuals was evidenced here contrasting with functional tests displaying weaker inter-individual variations. This feature raises several issues within the framework of individual susceptibility to pollutants.

Some of the proteins identified here are in agreement with previous research describing MeHg toxicity mechanisms leading to cell death. MeHg exposure is known to induce a rapid and sustained increase in intracellular calcium levels [79,80]. The earliest detectable event following MeHg exposure is a change in the level of mitochondria [81]. Exposure of T-Cells to MeHg chloride has been found to cause a decrease in the overall size of mitochondria and changes in the structure of the cristae, leading finally to apoptosis. [81,82]. A previous study observed the expression and activation of different caspases after 16 h of treatment with MeHg [82]. Caspases are cysteine proteases that are essential for executing apoptosis and for degrading vimentin [83,84]. The lower vimentin expression found here in exposed MeHg lymphocytes agrees with this caspase activation.

In this study, we found that exposure for 72 hours *in vitro *to 1 μM of MeHg affected not only cell proliferation but also many cellular functions such as the building of the cytoskeleton, melatonin biosynthesis, the creation of signal transduction pathways and signal transcription. This variability in affected cellular functions suggests various toxicity pathways, depending on duration of exposure, MeHg concentration, cell type and individual susceptibility. This study shows the potential of using a proteomics approach in deciphering the intracellular changes in cells exposed *in vitro *to MeHg.

## Conclusion

The T-Hg level analysed in the blood of wild harbour seals from the German North Sea are high compared to previous studies on marine mammals and humans. The T-Hg level observed reflects both seal piscivorous habits and the contamination of the North Sea. Our cell model revealed an *in vitro *immunosuppressive effect of MeHg, even at low concentrations (0.2 and 1 μM). Although the *in vitro *approach utilised in this investigation represents an extreme reductionism relative to the complex situation in the intact organism, it can provide an insight into specific effects of model agents. However, many questions are raised. One is the biological relevance of these *in vitro *phenomena. In this regard, concentrations of Hg that are active *in vitro *are comparable to those found in seal blood. A second question, related to the issue of potential biological relevance, is whether increased levels of mercury in seals are epidemiologically associated with immunological alterations (e.g. epizootics). This question is debatable, as the association between mercury and health is well documented for humans but is less evident for marine mammals, which are believed to be more resistant to Hg exposure. Our results suggest that seal and human PBMCs react in a comparable way to MeHg *in vitro *exposure with, however, larger inter-individual variations. MeHg could be an additional cofactor in the immunosuppressive pollutant cocktail generally described in the blood of seals and this therefore raises the possibility of additional additive effects in the marine mammal immune system.

## Abbreviations

T-Hg: Total mercury; MeHg: methylmercury; PBMBc: peripheral bloodmononuclear cells; PHA: phytohemaglutinin; GAPDH: glyceraldehyde-3-phosphate; IL-2: interleukin-2; IL-4: interleukin-4; TGF-β: Transforming Growth Factor-β; RT-PCR: Reverse Transcriptase Polymerase Chain Reaction; mRNA: messenger RiboNucleic Acid; inner salt, MTs assay: 3-(4,5-dimethylthiazol-2-yl)-5-(3-carboxymethoxyphenyl)-2-(4-sulphophenyl)-2H-tetrazolium; Mowse: MOlecularWeightSEarch; 2D DIGE: Two Dimension Difference Gel Electrophoresis; Cye: cyanine; μM: micromolar; PBS: phosphate-buffered saline; FW: fresh weight; PBMC: Peripheral Blood Mononuclear Cell.

## Competing interests

The authors declare that they have no competing interests.

## Authors' contributions

KD conceived of the study and participated in its design and coordination, provided expert advice on Hg exposure and drafted the manuscript. US participated in the study design, coordinated the sample collection and provided expert advice on seals. CD, AG and AD contributed to sample preparation and data acquisition. SF contributed to the study design and provided expert advice on cytokines. GM and EDP provided expert advice on proteomics. MCDPG participated in the study design, provided expert advice on cell culture and functional tests and contributed to the preparation of the manuscript. All authors read and approved the final manuscript.

## Supplementary Material

Additional file 1 **Table 4.** Expression of identified proteins after spot excision. Click here for file
