# Supplementary material for: Mercury immune toxicity in harbour seals: links to in vitro toxicity
Source: Environ Health. 2008 Oct 29;7:52. doi: 10.1186/1476-069X-7-52 (PMC2600635; doi:10.1186/1476-069X-7-52)
Supplement: Additional file 1 — Table 4. Expression of identified proteins after spot excision. [file 1476-069X-7-52-S1.doc]

**Table 4 : Expression of identified proteins after spot excision**

**From 3 individuals separately**

| **Spot number** | **Ref** | **Name** | **Abundance** | **Volume ratio** | **Mw (Da)** | Cellular function | **Mowse**  **score** |
| --- | --- | --- | --- | --- | --- | --- | --- |
| 1534 | P63244 | GBLP : Receptor of activated protein kinase C 1 | **↓** | -1.51 | 35380 | Signal transduction  . | 118 |
| 1601 | P39687 | AN32A :Acidic leucine-rich nuclear phosphoprotein 32 family member A | **↓** | -1.93 | 28682 | Signal transduction pathway | 62 |

*spot number after figure 9

**Pooled from 3 individuals**

| **Spot number *** | **Ref** | **Name** | **Abundance** | **Volume ratio** | **Mw (Da)** | Cellular function | **Mowse score** |
| --- | --- | --- | --- | --- | --- | --- | --- |
| 1142 | P23381 | SYW : Tryptophanyl-tRNA synthetase | ↑ | 1.52 | 53474 | Negative modulation of cell proliferation ; protein biosynthesis | 170 |
| 1319 | P08670 | VIME : Vimentine | ↓ | -1.67 | 53545 | Cytoskeletal protein | 286 |
| 1452 | P62333 | PRS10 : Proteasome 26S subunit ATPase 6 | ↑ | 1.63 | 44430 | Involved in the degradation of ubiquitineous proteins (ATP dependant). | 139 |
| 897 | O95671 | ASML : N-acetylserotonin O-methyltransferase-like protein | ↓ | -1.53 | 69526 | Melatonin biosynthesis and methyltransferase activity | 55 |
| 923 | Q9H0C5 | BTBD1 : BTB/POZ domain-containing protein 1 | ↓ | -1.55 | 53365 | Interact with topoisomerase 1 and isoform Delta of the ring finger protein TRIM5. | 55 |

*spot number after figure 10
